# Driver Stress Detection Using Ultra-Short-Term HRV Analysis under Real World Driving Conditions

**DOI:** 10.3390/e25020194

**Published:** 2023-01-19

**Authors:** Kun Liu, Yubo Jiao, Congcong Du, Xiaoming Zhang, Xiaoyu Chen, Fang Xu, Chaozhe Jiang

**Affiliations:** 1 School of Transportation & Logistics, Southwest Jiaotong University, Chengdu 610097, China; 2 School of Mines, China University of Mining and Technology, Xuzhou 221116, China; 3 Department of Aeronautical and Aviation Engineering, The Hong Kong Polytechnic University, Hung Hom, Hong Kong, China; 4 Department of Purchase Management, Sichuan Tourism University, Chengdu 610100, China

**Keywords:** driving safety, stress detection, heart rate variability, classification, machine learning

## Abstract

Considering that driving stress is a major contributor to traffic accidents, detecting drivers’ stress levels in time is helpful for ensuring driving safety. This paper attempts to investigate the ability of ultra-short-term (30-s, 1-min, 2-min, and 3-min) HRV analysis for driver stress detection under real driving circumstances. Specifically, the *t*-test was used to investigate whether there were significant differences in HRV features under different stress levels. Ultra-short-term HRV features were compared with the corresponding short-term (5-min) features during low-stress and high-stress phases by the Spearman rank correlation and Bland–Altman plots analysis. Furthermore, four different machine-learning classifiers, including a support vector machine (SVM), random forests (RFs), K-nearest neighbor (KNN), and Adaboost, were evaluated for stress detection. The results show that the HRV features extracted from ultra-short-term epochs were able to detect binary drivers’ stress levels accurately. In particular, although the capability of HRV features in detecting driver stress also varied between different ultra-short-term epochs, MeanNN, SDNN, NN20, and MeanHR were selected as valid surrogates of short-term features for driver stress detection across the different epochs. For drivers’ stress levels classification, the best performance was achieved with the SVM classifier, with an accuracy of 85.3% using 3-min HRV features. This study makes a contribution to building a robust and effective stress detection system using ultra-short-term HRV features under actual driving environments.

## 1. Introduction

Driver stress can affect a driver’s decision-making, performance, and perception abilities [1], increasing traffic safety risks [2]. One study estimated that 30 percent of road crashes are caused by driver stress, thus making stress a major contributor to crashes [3]. Prolonged exposure to driving stress can cause drivers to suffer from headaches, reduced sleep quality, and even increased cardiovascular risk [4]. Therefore, detecting driver stress is crucial for improving driver performance to ensure traffic safety.

Previous research mainly assessed driver stress, based on the driver’s psychological, physical, and physiological responses. Psychological assessments have been used as ground truth in stress detection in most studies. Questionnaires or self-reporting methods [5] are used to measure drivers’ stress after driving. However, conducting questionnaires at the end of the experiment may not reflect the driver’s feelings accurately, affecting the assessment results [6]. Some studies have attempted to detect driver stress by monitoring drivers’ physical responses, such as facial expressions [7] and vehicle dynamic data [8]. Additionally, researchers also have concentrated on physiological-based assessment methods, such as heart activity [9], electrodermal activity [6], and respiration activity [1]. Particularly, heart rate variability (HRV), which could be collected non-invasively, has been one of the prevalent methods to assess driver stress [10]. HRV describes the interval variation between consecutive R-wave peaks and can reflect the fluctuation of the autonomic nervous system (ANS), which directly affects cardiac activity. HRV signals are commonly analyzed in time-domain, frequency-domain, and non-linear analyses to detect driver stress. During stress phases, HRV features significantly vary with the fluctuations of the ANS activities, making them good indicators of drivers’ stress [9].

The recommended minimum duration for a short-term HRV analysis is 5 min, which has been used to detect driver stress in many studies [9,10,11]. Specifically, there is an outstanding performance for using short-term (5 min) HRV features to detect driver stress in real driving conditions [11]. However, fewer studies have investigated the estimation of the driver’s stress levels using an ultra-short-term (less than 5 min) HRV analysis. In fact, the epochs for a 5-min HRV analysis may be too long to detect driver stress in time, resulting in the failure for alerting the driver to avoid traffic incidents and accidents. To address this problem, many researchers have used an ultra-short-term HRV analysis, which could be used to monitor driver stress levels in a real-time and continuous manner, to detect driver stress levels [12,13]. Although those studies tried to use some HRV features extracted from less than 5-min epochs for driver stress detection, they ignored the difference in the stress detection abilities between various ultra-short-term and short-term HRV features. Moreover, previous studies conducted ultra-short-term HRV analyses for mental stress detection using the data collected in well-designed experiments, rather than those collected in the field [14] [15]. Considering that participants could feel more relaxed in the lab, resulting in different HRV feature changes between the data collected in the field and in the lab, conducting an ultra-short-term HRV analysis with data in the field may represent authenticity and have high value for practical applications. Moreover, ultra-short-term HRV analyses have been employed in various clinical fields [16,17]. Some studies have shown that ultra-short-term HRV features are valid surrogates for short-term HRV features for investigating the ANS function in obstructive sleep apnea (OSA) patients [18,19]. Although Munoz et al. [20] evaluated the validity of two HRV features extracted from 10-sec epochs, it is unclear to measure the stress level for other HRV features, since more HRV features could be used to build a more robust detection model. Therefore, there is a challenge to build a real-time and robust stress detection model using ultra-short-term HRV features.

In this study, we focus on driver stress detection and assessment using an ultra-short-term HRV analysis in real work conditions. Firstly, a statistical analysis was performed to determine which ultra-short-term HRV feature could be used to substitute the corresponding short-term HRV feature, to detect driver stress levels. Then, the machine learning approach was employed to build a classification model for stress detection. To the best of our knowledge, this is the first study to analyze driver stress detection using ultra-short-term HRV features in actual driving conditions. The ultra-short-term driver stress detection model could be used to monitor driver stress states in a short and real time manner, alerting the driver to make adjustments in a timely manner and thereby ensuring traffic safety.

## 2. Materials and Methods

### 2.1. Stress Data

**Figure 1:**
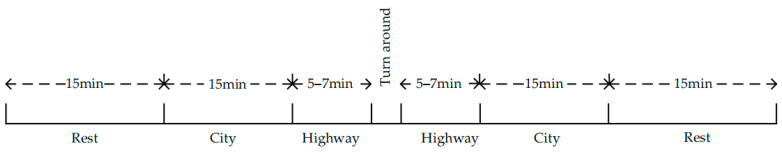
Overview of the experiment.

The data used in this study were the ECG signals in the driver database [1]. The data include the signals of 17 drivers, who were required to drive a prespecified route in Boston. As shown in Figure 1, the driving route contains three driving scenarios: rest, city-driving, and highway-driving, which were designed to induce drivers’ varying stress feelings. Specifically, two 15-min rest periods occurred at the beginning and end of the drive. During the rest periods, the driver sat in the garage with eyes closed to create a low-stress level. Following the first rest period, the drivers drove on a busy street with unexpected risks generated by non-motorized traffic flow, resulting in a high-stress level. The route then led drivers away from the city, over a bridge, and onto a highway. During highway driving, the drivers would have a medium-stress level when driving without congestion. Finally, drivers turned around and followed the same route in the opposite direction, back to the starting point. Each driving experiment lasted around 60 to 90 min, depending on the traffic conditions.

Three digital cameras were used to record video during the whole driving experiment, including the first camera placed at the steering wheel, the second wide-angle camera was mounted on the dashboard, and the third camera was used for event recording. Then, two experts watched those videos to score the driver’s stress level, according to some stress indicators (e.g., stops, turning, bumps in the road, head-turning, and gaze changes). Following the experiment, the drivers were required to complete subjective rating questionnaires immediately, including two main ratings: a subjective stress rating scale and a stressful event rating scale. The results from both expert ratings and subjective questionnaires validated the assumption that the experimental scenarios can induce defined stress levels: low stress, moderate stress, and high stress, corresponding to rest, highway, and city street driving [1].

### 2.2. Pre-Processing

**Figure 2:**
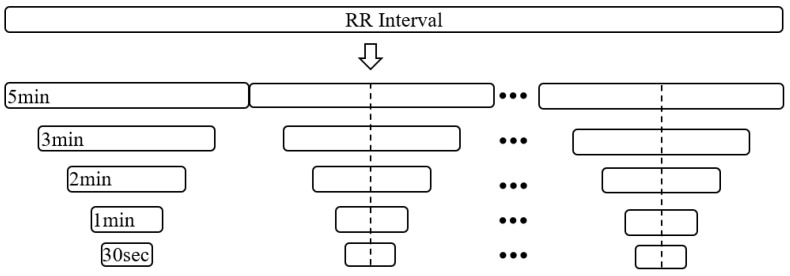
Cutting a 5-min epoch into some shorter epochs.

The RR intervals were extracted from the ECG signals using the PhysioNet HRV toolkit, a rigorously validated open-source software package for HRV analysis [21]. Moreover, outliers and ectopic beats were removed from those RR intervals. An outlier is defined as a point outside the range of 280 to 1500 milliseconds [22], and an ectopic beat is a point where the RR interval differs from the previous RR interval by more than 20% [23]. Both outliers and ectopic beats were replaced by interpolated intervals with linear interpolation [22]. Moreover, to analyze the HRV features extracted from various epochs, all RR intervals were divided into 5-min epochs without overlapping. Secondly, the shorter epochs (i.e., 30-s, 1-min, 2-min, and 3-min epochs) were obtained from the corresponding 5-min epoch. Specifically, 30-s, 1-min, 2-min, and 3-min epochs have the same center point with corresponding 5-min epochs, as shown in Figure 2.

The recordings from the first 5 min of each experiment were discarded due to a poor signal quality. Moreover, only “low-stress level” and “high-stress level” recordings were considered in this study to obtain a clearer distinction between the different stress levels.

### 2.3. HRV Features

For each epoch, 22 commonly used HRV features were extracted from the RR intervals, according to Table 1, including 10 time-domain features, seven frequency-domain features, and five non-linear features. The Lomb–Scargle periodogram, which is suitable for small sample RR intervals and does not require resampling, was used to analyze the power spectral density of the RR intervals.

### 2.4. Statistical Analysis

#### 2.4.1. Student’s *t*-Test

The *t*-test was conducted to investigate whether there is a statistically significant difference in those HRV features between the varying stress levels. Specifically, the *t*-test was performed for different time scales, respectively, to explore how the HRV features extracted from different time scales related to the stress levels. It is assumed that, for an HRV feature, there is the same ability of stress detection in all time scales, if a significant difference and the same tendency were found for it and extracted from the various time scales, between the varying stress levels. The significance level was set at *p* < 0.05.

#### 2.4.2. Spearman Correlation Analysis and Bland–Altman Plots

The Spearman correlation analysis, a nonparametric rank statistic, was carried out to determine whether an ultra-short-term HRV feature was correlated with the corresponding short-term HRV feature at the same stress level. Although Spearman’s rank correlation coefficient has been used to measure the degree of association between two variables, the strong correlation does not necessarily indicate an agreement. Considering that the Bland–Altman analysis could be used to measure the mean difference and calculate the limits of agreement(LoA) for assessing the agreement between two methods of measurement [25,26], the Bland–Altman analysis was further used to assess the agreement between the ultra-short HRV features and the corresponding short-term HRV features at the same stress level. It would be assumed that an ultra-short-term HRV feature could be used to replace the corresponding short-term HRV feature for driver stress detection, if an HRV feature meets the following conditions: (1) there are the same changes for all of the ultra-short-term HRV features and corresponding short-term HRV feature, between the low-stress and high-stress levels, and (2) there are significant correlations for all of the ultra-short-term HRV features and corresponding short-term HRV features between the low-stress and high-stress levels (i.e., the correlation coefficient is higher than 0.7, and the correlation coefficient is significantly different). The significance level of Spearman’s correlation coefficient was set at 0.05.

### 2.5. Stress Classification

**Figure 3:**
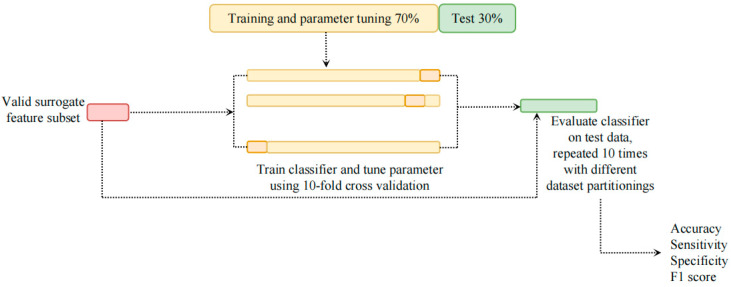
Flowchart of the classification steps.

The machine learning pipeline used in this work is shown in Figure 3. For the ultra-short-term HRV features, only those features, which could be substituted for the corresponding short-term HRV features, based on a statistical analysis, were used to build the machine-learning models. The feature data was split into two parts: 70% of the data for training and parameter tuning, and 30% of the data for testing. To make sure that the results are not just coincidental, this process was repeated ten times. The 10-fold cross-validation was performed to research the optimal parameters using a grid search at each split. The accuracy, sensitivity, specificity, and F1-score were calculated to evaluate the performance of the models. The final test results are the mean values across these ten repetitions.

Four different binary classifiers were considered: support vector machine (SVM) [27], random forests (RFs) [28], K-nearest neighbor (KNN) [29], and AdaBoost [30]. These classifiers were chosen, partly because they are different from each other in algorithmic logic, but also because they have been used to build stress detection in previous studies. The SVM used a Gaussian kernel function. The KNN was trained with K varies from 1 to 10 and used the Euclidean distance metric. The RFs used 50, 100, and 150 decision trees. The Adaboost classifier used the same decision trees as the RFs and a learning rate of 0.1. Except for these parameters, the default settings in the Python packages sklearn version 0.19.2 were used.

## 3. Results

### 3.1. Ultra-Short-Term HRV Features Analysis

The *t*-test results and the trends of the HRV features are reported in Table 2 and Table 3, respectively. For the 5-min epochs, 18 of the 22 HRV features showed statistically significant differences between the low-stress and high-stress levels. Fourteen of these 18 features increased significantly with the increased stress level, while the remaining four features were significantly decreased. Moreover, the results of the ultra-short-term HRV features analysis showed that there were the same significant differences and trends for one HRV feature extracted from different time scales between low-stress and high-stress levels, suggesting that varying epoch lengths might not affect the changes of the HRV features between the different stress levels.

The correlation analysis results for ultra-short HRV features are reported in Table 4. For the time-domain features, four features (MeanNN, SDNN, NN20, and MeanHR) showed high correlation coefficients for 30-s or 1-min epochs (i.e., ultra-short vs. short time-epoch per each feature). For the frequency-domain features, only HF showed a high correlation for 1-min epochs. However, there was no high correlation coefficient in the frequency-domain features computed in 30-s epochs. For the non-linear features, CVI and SampEn showed high correlation coefficients for 1-min epochs, while none of the non-linear features showed high correlation coefficients for 30-s epochs. Moreover, there are high correlation coefficients in all HRV features extracted from 2-min or 3-min epochs between the low-stress and high-stress levels.

**Figure 4:**
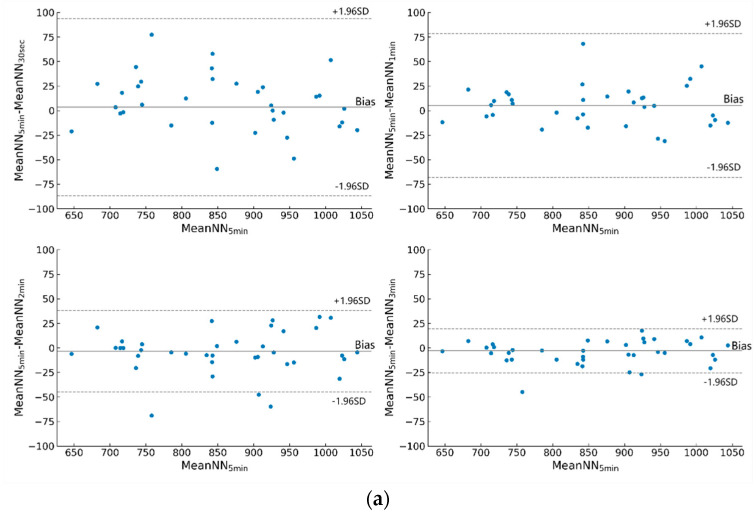

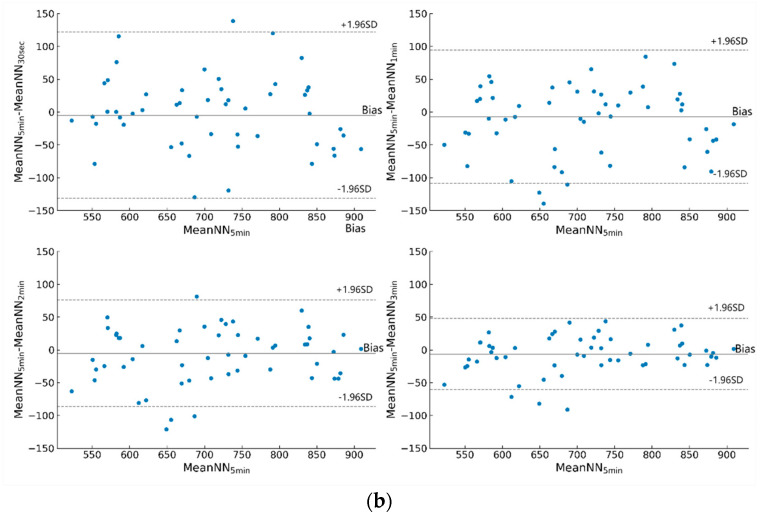
Bland–Altman Plots of MeanNN. (**a**) Bland–Altman Plots of MeanNN during high stress. (**b**) Bland–Altman Plots of MeanNN during low stress.

For 30-s epochs, MeanNN, SDNN, NN20, and MeanHR could be selected as valid surrogates for the short-term HRV features. For 1-min epochs, MeanNN, SDNN, NN20, MeanHR, HF, CVI, and SampEn were selected as valid surrogates for the short-term features for the driver stress detection. For 2-min or 3-min epochs, all the features, except for SDSD, pNN20, RMSSD, and SD1, could be used as surrogates for the short-term HRV features. For all time scales, MeanNN, SDNN, NN20, and MeanHR showed statistically significant differences between the low-stress and high-stress levels and high correlation coefficients across time-scales (i.e., each ultra-short vs. short time-scale per each feature). Therefore, MeanNN, SDNN, NN20, and MeanHR were selected as valid surrogates for the short-term HRV features to detect drivers’ stress levels. Furthermore, the results of the correlation analysis were supported by the visual inspection of the Bland–Altman plots. As the epoch length increases, a decrease in bias and in width of the 95% LoA (±1.96 SD) was observed. The Bland–Altman plots analyses of MeanNN are shown in Figure 4.

### 3.2. Stress Classification with Ultra-Short-Term HRV Features

For the feature selection, we selected four HRV features, which were valid surrogates for the short HRV features across all time scales, as input to build the driver’s stress detection model. Since the 5-min epoch is recommended for short-term HRV analyses, the performance of various classifiers with different ultra-short-term HRV features was compared with those classifiers with 5-min HRV features. Table 5 shows the performance of different classifiers using four short-term HRV features (i.e., MeanNN, SDNN, NN20, and MeanHR) as input. The best classification performance was achieved with the SVM classifier, whose mean accuracy was 87.5%.

The best-performing classifier was evaluated in various time scale settings. Hence, the SVM classifier with those HRV features (i.e., MeanNN, SDNN, NN20, and MeanHR) was evaluated for each time scale. The results are summarized in Table 6. The best classification accuracy was achieved with 3-min epochs, whose accuracy was 85.3%, while the accuracy of the SVM classifier using 30-s epochs was 85.0%. Performance dropped by about 3 percentage points using ultra-short-term HRV features, compared with short-term HRV features. Although the HRV features extracted from different time scales as input data had an impact on the performance of stress classification, the ultra-short-term HRV features could still detect drivers’ stress levels with a good performance.

## 4. Discussion

In this study, we investigate whether ultra-short-term HRV features could be used to measure and assess drivers’ stress. Considering that a 5-min HRV analysis may be too long to detect driver stress in time, an ultra-short-term (less than 5 min) HRV analysis was conducted. The *t*-test results for the HRV features showed that almost all HRV features extracted from the 5-min epochs had statistically significant differences between the low-stress and high-stress levels. The significant differences in ultra-short-term HRV features under different stress levels were in accordance with 5-min HRV features. Moreover, the tendency of ultra-short-term HRV features is the same as the tendency of the corresponding short-term HRV features between various stress levels, which are consistent with another study’s findings [31].

For the correlation analysis, the HRV features extracted from 2-min or 3-min epochs had high correlation coefficients with short-term HRV features. In particular, all frequency-domain HRV features extracted from 2-min or 3-min epochs were strongly correlated with those HRV features extracted from 5-min epochs, which is consistent with the findings of another study [32]. Considering that at least a 1-min length is required to calculate the HF band and at least a 2-min length is required to estimate the LF band [32], the results show that LF had a very low Spearman coefficient, below 2 min, whilst for HF, it was below 1 min. Moreover, a 5-min length is required to evaluate the VLF band [33], but in this study, the VLF calculated from 5-min epochs had a high strong correlation for the ultra-short-time features extracted from over 2-min epochs. Regarding the non-linear HRV features, few studies have investigated their reliability in ultra-short-term lengths. Moreover, the correlation coefficient between the ultra-short-term and short-term HRV features decreased with the decreased length of the ultra-short-term epochs. Moreover, MeanNN, SDNN, NN20, and MeanHR extracted from various time scales have high correlation coefficients with those extracted from 5-min epochs, suggesting that those HRV features could be good surrogates for the short-term HRV features to detect drivers’ stress levels.

For driver stress detection, the results show that it is feasible to detect drivers’ stress levels in a very short time. The performances of four different classifiers were evaluated for stress detection with ultra-short-term HRV features. Although the best classification performance was achieved by the SVM classifier using short-term HRV features as input with an accuracy of 87.5%, the performance of the SVM classifier using ultra-short-term HRV features as input was not bad. The performance dropped by about 3 percentage points for the stress detection with ultra-short-term HRV features, suggesting that the ultra-short-term HRV features could be good surrogates for the short-term HRV features for stress detection. Moreover, previous studies have used the driver dataset used in this study to explore the stress detection method. Our model’s performance is superior to that of those studies. Munla et al. [9] used an SVM-RBF classifier using 5-min epochs as input to predict driver stress levels and achieved an accuracy of 83%, which is lower than the accuracy achieved in our study. Dalmeida et al. [12] proposed a method to automatically detect stress with HRV features computed by 30-s epochs and obtained an accuracy of 85% (sensitivity = 81% and F1-score = 78%). However, it does not explore that ultra-short-term features are significantly correlated with 5-min features. The redundant features may affect the model’s performance. Our study has confirmed that not all 30-s HRV features are a good substitute for 5-min HRV features. Moreover, Vargas et al. [34] analyzed the ability of EMG signals with different time scales (1 min, 2 min, 3 min, 4 min, and 5 min) for driver stress detection. Their results showed that an ultra-short-term EMG analysis was not able to detect drivers’ stress levels, and the accuracy of the ultra-short-term epochs dropped by about 20%, compared to short-term epochs. However, none of the studies have focused on ultra-short-term HRV analyses for driver stress detection.

Our result indicated that MeanNN, SDNN, NN20, and MeanHR could be used to replace the corresponding short-term HRV feature to detect driver stress levels. Moreover, to explore the ability of the single features to detect drivers’ stress, each valid surrogate feature using 5-min epochs was used, respectively, as input for the classifiers. The performances of these classifiers dropped by about 10–15 percentage points using each valid surrogate feature as input, compared with the combination of these HRV features.

While this study provides stress detection with ultra-short-term HRV features, important limitations are noted. Although the stress detection model in this study was developed, based on some ultra-short-term HRV features (i.e., MeanNN, SDNN, NN20, and MeanHR), more frequency-domain and non-linear HRV features should be explored for stress detection. More HRV features used as inputs might improve the models’ performance. Furthermore, the variation in cardiac activity between individuals could be considerably large, which may be influenced by age, weight, and gender. It has been shown that accounting for inter-individual variability may improve the classification performance [35]. Future work should investigate more powerful and robust models to accurately detect drivers’ stress, combining ultra-short-term HRV analysis and other physiological signals.

## 5. Conclusions

This study investigated the ability of ultra-short-term HRV analysis to detect and assess driver stress levels under real world driving conditions. Although the ability of short-term HRV analysis has been found for stress detection, the feasibility of stress detection using ultra-short-term HRV features has not been explored. Our study demonstrates that not all ultra-short HRV features are good surrogates of corresponding short-term HRV features. In particular, HRV features which could be used for stress assessment are different for different ultra-short-term time scales. Moreover, although ultra-short-term HRV features have a weak negative impact on the classification performance, compared with short-term HRV features, the performance of ultra-short-term HRV features still could reach a high level.

Driver stress detection, based on ultra-short-term HRV analysis, remains an interesting and challenging issue, which have not been addressed appropriately. With the advance in vehicle equipment, it is very important to detect drivers’ stress levels in time. The ultra-short-term HRV analysis could detect driver stress levels accurately and timely, alerting the driver to avoid traffic incidents and accidents. The findings of this study could contribute to building a robust and effective stress detection system using ultra-short-term HRV features for driver stress measurement and assessment in a short and real-time manner, which could be used to alert the driver to make an adjustment and thereby ensure traffic safety.

## Figures and Tables

**Table 1:** HRV features and their description.

Domain	HRV Feature	Unit	Description
Time	MeanNN	ms	Mean RR interval
	SDNN	ms	Standard deviation of the RR intervals
	SDSD	ms	The standard deviation of the differences between adjacent RR intervals
	NN50		Number of pairs of differences between adjacent RR intervals differing by more than 50 milliseconds
	pNN50		NN50 count divided by the total number of all RR intervals
	NN20		Number of pairs of differences between adjacent RR intervals differing by more than 20 milliseconds
	pNN20		NN20 count divided by the total number of all RR intervals
	RMSSD	ms	Square root of the mean of the sum of the squares of the differences between adjacent RR intervals
	MeanHR	bpm	Mean heart rate
	SDHR	bpm	Standard deviation of the heart rate
Frequency	LF	ms^2^	Power of the low frequency band (0.04–0.15 Hz)
	HF	ms^2^	Power of the high frequency band (0.15–0.4 Hz)
	LF/HF		Ratio of the LF to HF
	TP	ms^2^	Total power of the frequency band (≤0.4 Hz)
	VLF	ms^2^	Power of the very low frequency band (≤0.04 Hz)
	LFnu	nu	LF power in normalized units
	HFnu	nu	HF power in normalized units
Non-linear	CSI		Cardiac sympathetic index [24]
	CVI		Cardiac vagal index [24]
	SD1	ms	The standard deviation of the projection of the Poincaré plot on the line perpendicular to the line (y = x)
	SD2	ms	The standard deviation of the projection of the Poincaré plot on the line (y = x)
	SampEn		Sample entropy, which is a measure of complexity for the HRV time series data

**Table 2:** *t*-test results (*p*-value) for the HRV features between the low-stress and high-stress levels.

HRV Feature	30-s	1-min	2-min	3-min	5-min
MeanNN	**<0.001**	**<0.001**	**<0.001**	**<0.001**	**<0.001**
SDNN	**<0.001**	**<0.001**	**<0.001**	**<0.001**	**<0.001**
SDSD	0.061	0.120	0.095	0.083	0.306
NN50	**0.004**	**<0.001**	**<0.001**	**<0.001**	**<0.001**
pNN50	**0.001**	**<0.001**	**<0.001**	**<0.001**	**<0.001**
NN20	**<0.001**	**<0.001**	**<0.001**	**<0.001**	**<0.001**
pNN20	0.111	0.196	0.210	0.299	0.119
RMSSD	0.061	0.120	0.095	0.083	0.306
MeanHR	**<0.001**	**<0.001**	**<0.001**	**<0.001**	**<0.001**
SDHR	**<0.001**	**<0.001**	**<0.001**	**<0.001**	**<0.001**
LF	**<0.001**	**<0.001**	**<0.001**	**<0.001**	**<0.001**
HF	**<0.001**	**<0.001**	**<0.001**	**<0.001**	**<0.001**
LF/HF	**0.046**	**0.031**	**0.013**	0.067	**0.020**
TP	**<0.001**	**<0.001**	**<0.001**	**<0.001**	**<0.001**
VLF	**0.003**	**0.011**	**0.001**	**<0.001**	**<0.001**
LFnu	**0.001**	**0.002**	**0.001**	**0.006**	**0.001**
HFnu	**0.001**	**0.002**	**0.001**	**0.006**	**0.001**
CSI	**<0.001**	**<0.001**	**<0.001**	**<0.001**	**<0.001**
CVI	**<0.001**	**<0.001**	**<0.001**	**<0.001**	**<0.001**
SD1	0.070	0.129	0.100	0.086	0.311
SD2	**<0.001**	**<0.001**	**<0.001**	**<0.001**	**<0.001**
SampEn	**<0.001**	**<0.001**	**<0.001**	**<0.001**	**<0.001**

Note: Statistically significant effects (*p* < 0.05) between the stress levels are in bold.

**Table 3:** The trends of the HRV features’ mean.

HRV Feature	30-s	1-min	2-min	3-min	5-min
MeanNN	↓↓	↓↓	↓↓	↓↓	↓↓
SDNN	↑↑	↑↑	↑↑	↑↑	↑↑
SDSD	↑	↑	↑	↑	↑
NN50	↑↑	↑↑	↑↑	↑↑	↑↑
pNN50	↓↓	↓↓	↓↓	↓↓	↓↓
NN20	↑↑	↑↑	↑↑	↑↑	↑↑
pNN20	↓	↓	↓	↓	↓
RMSSD	↑	↑	↑	↑	↑
MeanHR	↑↑	↑↑	↑↑	↑↑	↑↑
SDHR	↑↑	↑↑	↑↑	↑↑	↑↑
LF	↑↑	↑↑	↑↑	↑↑	↑↑
HF	↑↑	↑↑	↑↑	↑↑	↑↑
LF/HF	↑↑	↑↑	↑↑	↑↑	↑↑
TP	↑↑	↑↑	↑↑	↑↑	↑↑
VLF	↑↑	↑↑	↑↑	↑↑	↑↑
LFnu	↑↑	↑↑	↑↑	↑↑	↑↑
HFnu	↓↓	↓↓	↓↓	↓↓	↓↓
CSI	↑↑	↑↑	↑↑	↑↑	↑↑
CVI	↑↑	↑↑	↑↑	↑↑	↑↑
SD1	↑	↑	↑	↑	↑
SD2	↑↑	↑↑	↑↑	↑↑	↑↑
SampEn	↓↓	↓↓	↓↓	↓↓	↓↓

Note: ↑↑ (or ↓↓) indicates significantly increased (or decreased) with increased stress level (*p* < 0.05); ↑ (or ↓) indicates increased (or decreased) with increased stress level (*p* > 0.05).

**Table 4:** Spearman correlation analysis of the HRV features during the low-stress and high-stress levels.

HRV Features	Low Stress				High Stress			
	30-s vs. 5-min	1 vs. 5-min	2 vs. 5-min	3 vs. 5-min	30-s vs. 5-min	1 vs. 5-min	2 vs. 5-min	3 vs. 5-min
MeanNN	**0.912** *	**0.945** *	**0.969** *	**0.989** *	**0.852** *	**0.887** *	**0.922** *	**0.962** *
SDNN	**0.793** *	**0.727** *	**0.841** *	**0.920** *	**0.760** *	**0.780** *	**0.908** *	**0.968** *
SDSD	**0.744** *	**0.852** *	**0.917** *	**0.974** *	0.414	0.571	**0.800** *	**0.908** *
NN50	0.565	**0.739** *	**0.840** *	**0.924** *	0.522	0.682	**0.753** *	**0.872** *
pNN50	0.595	0.682	**0.873** *	**0.953** *	0.524	0.694	**0.884** *	**0.918** *
NN20	**0.745** *	**0.803** *	**0.911** *	**0.950** *	**0.728** *	**0.730** *	**0.839** *	**0.915** *
pNN20	0.564	0.673	**0.870** *	**0.944** *	0.490	0.516	**0.707** *	**0.839** *
RMSSD	**0.747** *	**0.854** *	**0.917** *	**0.974** *	0.416	0.571	**0.800** *	**0.908** *
MeanHR	**0.890** *	**0.967** *	**0.980** *	**0.993** *	**0.805** *	**0.877** *	**0.916** *	**0.966** *
SDHR	0.503	0.653	**0.783** *	**0.880** *	0.542	**0.762** *	**0.837** *	**0.960** *
LF	0.495	0.692	**0.886** *	**0.943** *	0.546	**0.783** *	**0.901** *	**0.952** *
HF	0.527	**0.798** *	**0.769** *	**0.917** *	0.563	**0.723** *	**0.841** *	**0.940** *
LF/HF	0.576	**0.758** *	**0.922** *	**0.953** *	0.344	0.520	**0.740** *	**0.863** *
TP	0.532	0.679	**0.795** *	**0.888** *	0.584	**0.775** *	**0.905** *	**0.966** *
VLF	0.289	0.510	**0.727** *	**0.900** *	0.518	0.649	**0.819** *	**0.937** *
LFnu	0.576	**0.758** *	**0.922** *	**0.953** *	0.344	0.520	**0.740** *	**0.863** *
HFnu	0.576	**0.758** *	**0.922** *	**0.953** *	0.344	0.520	**0.740** *	**0.863** *
CSI	0.483	0.681	**0.848** *	**0.887** *	0.613	**0.734** *	**0.874** *	**0.948** *
CVI	0.596	**0.756** *	**0.854** *	**0.932** *	0.559	**0.754** *	**0.894** *	**0.962** *
SD1	**0.745** *	**0.854** *	**0.917** *	**0.974** *	0.415	0.572	**0.801** *	**0.908** *
SD2	0.521	0.673	**0.792** *	**0.913** *	0.571	**0.776** *	**0.909** *	**0.965** *
SampEn	0.608	**0.736** *	**0.899** *	**0.950** *	0.587	**0.739** *	**0.858** *	**0.907** *

Note: Statistically significant effects (*p_ρ_* < 0.05) are denoted by *: Spearman’s correlation coefficient (*ρ* > 0.7) is in bold.

**Table 5:** The accuracy, sensitivity, specificity, and F1-score for different classifiers using short-term HRV features.

Classifier	Accuracy Mean ± SD (Range)	Sensitivity Mean ± SD (Range)	Specificity Mean ± SD (Range)	F1-Score Mean ± SD (Range)
KNN	85.0 ± 6.3 (73.3–90.7)	89.3 ± 6.5 (77.0–93.8)	77.8 ± 9.2 (66.7–89.9)	87.7 ± 5.3 (77.8–95.6)
SVM	87.5 ± 4.5 (81.3–94.7)	86.0 ± 6.1 (77.8–93.4)	91.3 ± 3.6 (85.7–98.4)	89.7 ± 4.0 (84.8–97.2)
RF	81.0 ± 4.2 (73.3–86.7)	83.0 ± 7.3 (75.0–87.9)	78.8 ± 10.3 (58.3–90.9)	84.2 ± 3.4 (78.9–89.5)
Adaboost	82.7 ± 9.1 (66.7–93.3)	86.2 ± 10.7 (65.0–93.4)	77.7 ± 14.3 (41.7–90.9)	82.7 ± 9.1 (72.2–95.0)

**Table 6:** The performances of the SVM classifier using ultra-short-term HRV features.

Epoch	Accuracy Mean ± SD (Range)	Sensitivity Mean ± SD (Range)	Specificity Mean ± SD (Range)	F1-Score Mean ± SD (Range)
30-s	85.0 ± 7.4 (75.0–95.3)	82.8 ± 11.7 (55.6–88.2)	81.7 ± 13.1 (60.0–90.1)	86.6 ± 7.6 (68.9–95.4)
1-min	84.3 ± 5.2 (75.4–88.3)	78.0 ± 8.6 (71.2–87.5)	86.1 ± 8.1 (76.4–94.7)	82.1 ± 5.4 (68.8–87.2)
2-min	84.7 ± 5.3 (76.0–89.0)	83.8 ± 8.7 (69.0–94.7)	72.4 ± 7.0 (60.0–81.8)	82.4 ± 5.2 (72.7–90.0)
3-min	85.3 ± 4.1 (78.7–92.0)	78.1 ± 6.4 (66.7–87.5)	91.3 ± 3.6 (85.7–92.7)	85.2 ± 4.1 (77.4–91.4)

## Data Availability

Authors did not collect data from humans or animals. Data used in this research are from publicly available sources (https://physionet.org/content/drivedb/1.0.0/).
